# Infiltrating T lymphocytes and tumor microenvironment within cholangiocarcinoma: immune heterogeneity, intercellular communication, immune checkpoints

**DOI:** 10.3389/fimmu.2024.1482291

**Published:** 2025-01-08

**Authors:** Yunyan Dai, Chenyang Dong, Zhiming Wang, Yunpeng Zhou, Yi Wang, Yi Hao, Pinggui Chen, Chaojie Liang, Gaopeng Li

**Affiliations:** ^1^ Third Hospital of Shanxi Medical University, Shanxi Bethune Hospital, Shanxi Academy of Medical Sciences, Tongji Shanxi Hospital, Taiyuan, China; ^2^ First Clinical Medical College, Shanxi Medical University, Taiyuan, China; ^3^ Department of Nuclear Medicine, Nanyang First People’s Hospital, Nanyang, Henan, China; ^4^ Department of biliary and Pancreatic Surgery, First Hospital of Shanxi Medical University, Taiyuan, China; ^5^ Department of Hepatobiliary Surgery, Shanxi Bethune Hospital, Shanxi Academy of Medical Sciences, Third Hospital of Shanxi Medical University, Tongji Shanxi Hospital, Taiyuan, China

**Keywords:** tumor-infiltrating T lymphocytes, cholangiocarcinoma, tumor microenvironment, immune checkpoints, immunotherapy

## Abstract

Cholangiocarcinoma is the second most common primary liver cancer, and its global incidence has increased in recent years. Radical surgical resection and systemic chemotherapy have traditionally been the standard treatment options. However, the complexity of cholangiocarcinoma subtypes often presents a challenge for early diagnosis. Additionally, high recurrence rates following radical treatment and resistance to late-stage chemotherapy limit the benefits for patients. Immunotherapy has emerged as an effective strategy for treating various types of cancer, and has shown efficacy when combined with chemotherapy for cholangiocarcinoma. Current immunotherapies targeting cholangiocarcinoma have predominantly focused on T lymphocytes within the tumor microenvironment, and new immunotherapies have yielded unsatisfactory results in clinical trials. Therefore, it is essential to achieve a comprehensive understanding of the unique tumor microenvironment of cholangiocarcinoma and the pivotal role of T lymphocytes within it. In this review, we describe the heterogeneous immune landscape and intercellular communication in cholangiocarcinoma and summarize the specific distribution of T lymphocytes. Finally, we review potential immune checkpoints in cholangiocarcinoma.

## 1 Introduction

**Figure 1:**
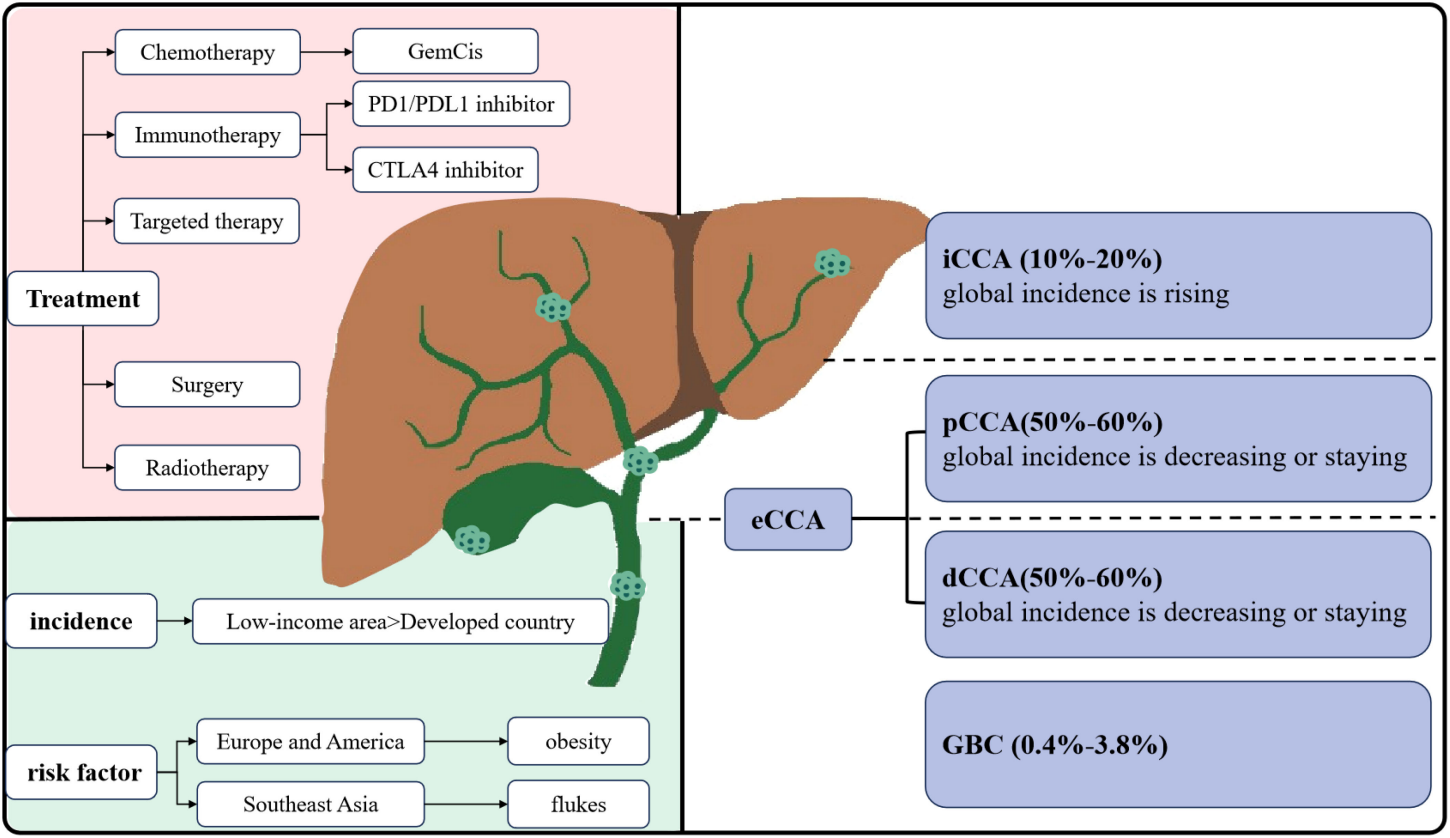
Classification and main treatment of CCA.

Biliary tract cancers (BTC) include cholangiocarcinoma (CCA) and gallbladder carcinoma (GBC) (1). CCA is further categorized into intrahepatic (iCCA), perihilar (pCCA), and distal (dCCA) cholangiocarcinoma based on its anatomical location within the biliary tree. Among these subtypes, pCCA is the most prevalent, accounting for 50–60% of CCA, followed by dCCA at 20–30%, and iCCA at 10–20% (2) (**Figure 1**).

CCA is acknowledged as a relatively rare form of cancer, comprising less than 2% of all cancers (3). A epidemiological analysis in the United States indicated that the incidence of CCA, iCCA, and extrahepatic cholangiocarcinoma (eCCA) has increased by 43.8%, 148.8%, and 7.5%, respectively (4). A study conducted in European countries revealed that mortality rates due to iCCA are rising at a higher rate than those due to eCCA (5). Additionally, while increasing mortality from iCCA has been observed globally, mortality from eCCA has either leveled off or decreased (6). CCA accounts for nearly 15% of all primary hepatic carcinomas and 3% of gastrointestinal cancers (5). The incidence and etiology of CCA subtypes vary across different regions. Developed regions exhibit low incidences of CCA, with rates falling below 2 per 100,000 (7). In the Western world, primary sclerosing cholangitis, metabolic syndrome, and nonalcoholic fatty liver disease are recognized risk factors for iCCA (8). In contrast, low-income countries in Southeast Asia report significantly higher incidences of CCA, up to 40 times that of Western countries. Infections with specific flukes have been identified as the primary cause of CCA in endemic regions, such as Thailand and China (9). In conclusion, the complex etiology and regional variations in incidence are closely linked to the highly heterogeneous subtypes of CCA.

Multiple diagnoses and distinct therapies for CCA are contingent on specific genetic aberrations and the primary site of the disease (10). Most CCAs are asymptomatic, with diagnosis is usually made at more advanced stages. Regional lymph node invasion is present in nearly half of patients, and distant metastases affect approximately one-quarter (11). This contributes to poor prognosis, high mortality rates, and limited treatment options, with a 5-year mortality rate of 80% (12). Surgical resection remains the preferred treatment for localized disease. Unfortunately, due to delayed diagnosis and locally advanced situation, curative resection is possible in less than 30% of patients (13). Even patients who undergo potentially curative surgical resection experience a high rate of recurrence and early local or distant metastases. Currently, the combination of gemcitabine and cisplatin (GemCis) is considered the standard treatment for unresectable or metastatic CCA (14), and ongoing clinical trials are investigating various targeted therapies. Nevertheless, dismal survival rates and adverse side effects following chemotherapy significantly affect patients’ quality of life. In summary, overall survival and genuine benefit of surgical resection and adjuvant therapy for CCA remain suboptimal (15, 16).

Immunotherapy has demonstrated significant potential in the treatment of solid tumors by effectively enhancing antitumor immunity through the modulation of immune checkpoints (17). The TOPAZ-1 trial, which evaluated the combination of GemCis and durvalumab in cholangiocarcinoma patients, has shown a notable improvement in overall survival (OS) and progression-free survival (PFS) for those with advanced cholangiocarcinoma (18). However, grade 3-4 adverse events were reported in three-quarters of patients, and the overall health of the patients demonstrated a prolonged trend of deterioration. Additionally, the study also did not reveal the influence of PD-L1 expression, primary tumor site, disease state, or geographic region on the findings. The positive results of the KEYNOTE-966 trial, a Phase III clinical study assessing Pembrolizumab in combination with chemotherapy for advanced biliary tract tumors, provided additional evidence supporting the incorporation of immune checkpoint inhibitors into standard chemotherapy regimens (19). Based on these two trials, the role of immune checkpoint inhibitors (ICIs) in the first-line treatment of advanced CCA is firmly established. However, the efficacy of ICIs in unselected groups of patients with advanced CCA is limited. Therefore, identifying predictive biomarkers for patients and understanding their resistance mechanisms are critical (20).

## 2 The characteristics of tumor microenvironment in cholangiocarcinoma

The tumor microenvironment (TME) constitutes a complex microecosystem surrounding a developing and progressing tumor. It includes not only the tumor cells themselves but also cancer-associated fibroblasts, vascular endothelial cells, and immunocytes from both innate and adaptive immune systems. Additionally, the TME comprises an extracellular matrix rich in various proteins such as collagen, laminin, and proteoglycan complexes (21, 22). The formation of this highly dynamic, multicellular functional compartment in conjunction with tumor growth is a hallmark feature of numerous epithelial cancers, which are often characterized by significant invasiveness and limited therapeutic options (23).

### 2.1 The phenotypic conversion and functional changes of tumor-infiltrating cells

In this internal environment, cancer-associated fibroblasts (CAFs) are the primary cells that contribute to tumorigenesis and are likely involved in tumor progression (24). CAFs may induce immune exclusion by overproducing aberrant extracellular matrix (ECM) (25), thereby affecting the immune microenvironment and delivery of chemotherapy drugs (26). As cancer advances, all cells undergo phenotypic conversion and functional changes (27). In innate immune cells, tumor-associated macrophages (TAMs) consistently tend to differentiate into the M2 phenotype, which possesses protumorigenic characteristics. The current conflicting findings in studies evaluating TAMs and patient outcomes in CCA suggest the need for further exploration of the relationship between TAMs and mechanisms underlying CCA progression (28, 29). Tumor-associated neutrophils (TANs) are inflammatory during the early stages of tumor development but adopt an immunosuppressive phenotype as the tumor progresses (30). Natural killer cells (NKs) are recognized for their potent cytotoxic effector functions, their ability to eliminate malignant cells and limit tumor metastasis is constrained within the TME (31). Some studies have indicated that the low cytotoxic activity of NKs is associated with an increased risk of cancer (32). Dendritic cells play a crucial role in maintaining communication between adaptive and innate immune cells, and are essential for orchestrating specific antitumor immune responses (33). The ability of tumor-infiltrating dendritic cells (TIDCs) to efficiently process antigens may be suppressed in the TME, but this capability can be restored by exiting this immunosuppressive milieu (34).

Over the past decade, numerous studies have demonstrated the critical role of adaptive immune cells in the antitumor immune response (35–37), with particular emphasis on T lymphocytes, which will be discussed in greater detail later. Similar to tumor-infiltrating T cells, tumor-infiltrating B cells (TIL-Bs) are adaptive immunocytes with diverse functions. In addition to their pro-tumorigenic effects, B cells exhibit antitumor activity (38). In the TME, CD8^+^ T lymphocytes, CD4^+^ T lymphocytes, and NKs are activated to block tumor propagation and inhibit immune escape. Conversely, other immunocytes such as DCs, regulatory T cells (Tregs), and TAMs promote tumor growth, progression, invasion, and angiogenesis, thereby inhibiting the antitumor immune response (39).

### 2.2 The intercellular communication and disease prognosis in cholangiocarcinoma

**Figure 2:**
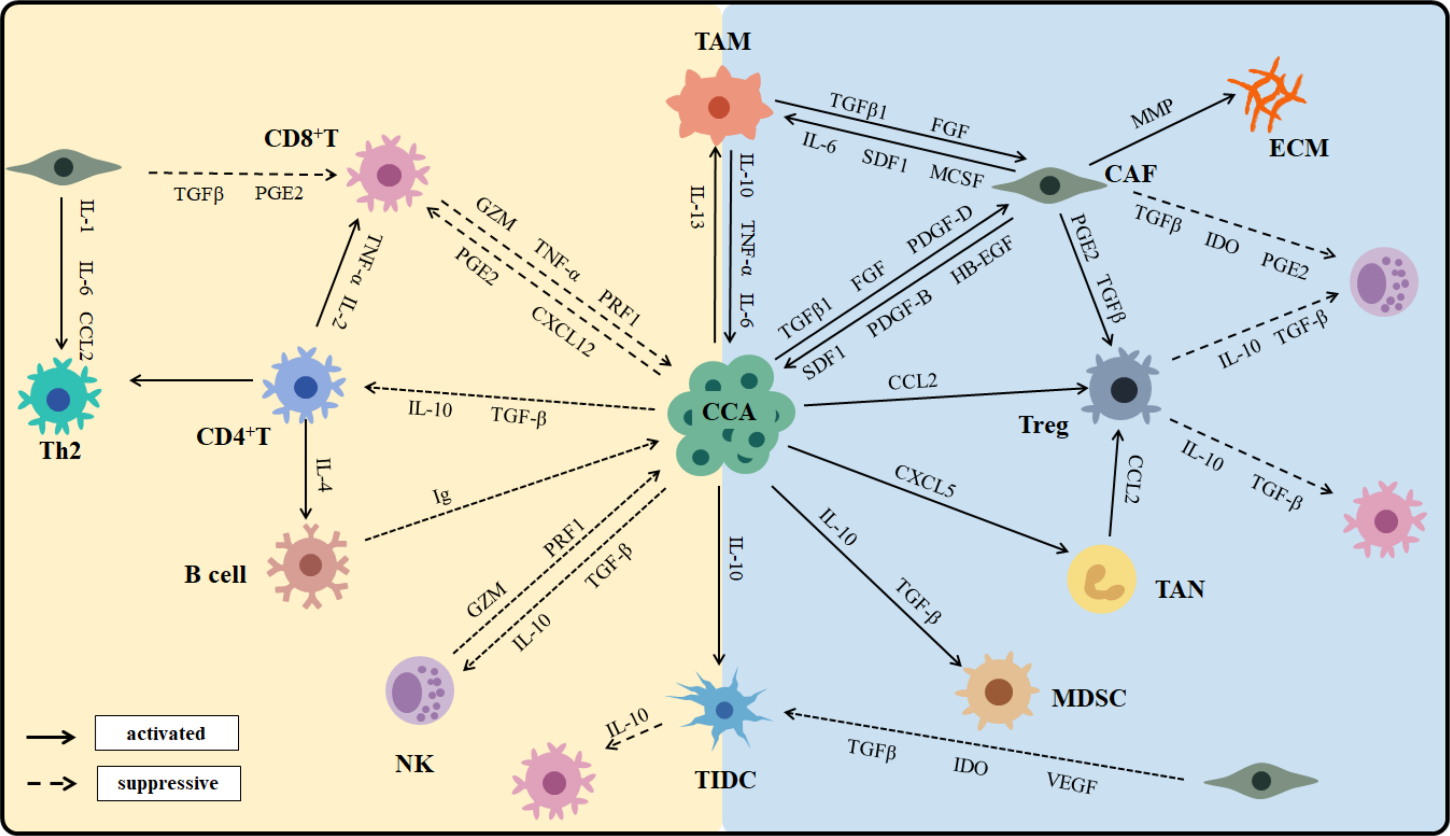
The cross-talk and interaction of immune cells in CCA.

CCA is characterized by a desmoplastic tumor microenvironment that encompasses a complex immunological landscape and a tumor-reactive stroma. The tumor microenvironment of CCA is notably enriched in myeloid cells, particularly TAMs and TANs, along with other immunosuppressive populations (40–42). In contrast, cells that mediate antitumor immunity are markedly diminished (43). The CCA phenotype is shaped not only by epigenetic alterations within the cancer cells but also by extensive crosstalk between malignant cells and their surrounding cellular environment (44). Cell–cell communication in CCA generates and maintains an immunosuppressive environment; tumors typically reprogram the TME to support survival (21) (**Figure 2**).

CAFs generate an extracellular matrix (ECM) that provides immune barriers, contributing to the highly desmoplastic tumor microenvironment characteristic of CCA (45). CAFs also secrete heparin-binding epidermal growth factor (HB‐EGF), which activates the epidermal growth factor receptor (EGFR) expressed by CCA cells (46). Additionally, CAFs attract DCs and dampen the expression of antigen-presenting molecules, which impair their ability to activate tumor-infiltrating lymphocytes (TILs) and stimulate immunosuppressive functions (47). Among all innate immune cells, TAMs represent the most significant population within the TME (48). CCA cells induce polarization of macrophages toward the M2 phenotype via the STAT3 pathway. TAMs participate in tumor growth and metastasis by releasing TNF-α, TGF‐β, IL6, IL10, and VEGF-A. High infiltration of TAMs is associated with angiogenesis and increased recruitment of Tregs and has been linked to poor prognosis in CCA patients. TANs are predominantly driven by C-X-C motif ligand 5 (CXCL5) and express CCL2 and CCL17, which recruit TAMs and Tregs, ultimately creating an immunosuppressive environment that sustains CCA progression (49). An increased presence of TANs in the TME, along with an elevated preoperative peripheral blood neutrophil-to-lymphocyte ratio, are poor prognostic factors for CCA.

NKs are recruited into the TME by CXCL9, where they utilize death receptor-mediated apoptosis and perforin/granzyme-mediated cytotoxicity to target tumor cells and inhibit primary tumor growth (50). Preclinical and clinical studies have shown that NK cell deficiency or impaired function is associated with an increased incidence of various malignancies. TIL-Bs contribute to limiting tumor growth by secreting immunoglobulins, enhancing T cell responses, and directly destroying cancer cells (51). Although TIL-Bs constitute a minor proportion of TILs in cholangiocarcinoma, a higher density of infiltrating B cells is significantly correlated with longer PFS and OS in CCA patients (52).

### 2.3 The heterogeneity of the immune microenvironment in cholangiocarcinoma

The immune heterogeneity of the TME is a prevalent characteristic of cholangiocarcinoma (53), as demonstrated by variations in the abundance and composition of infiltrating immune cells, along with the diverse activation states observed within individual immune cell subtypes (54–56). A comprehensive understanding of TME heterogeneity is essential for elucidating the molecular and cellular landscape of immune cells in CCA, deciphering the varied responses to anti-tumor therapies among CCA patients, and facilitating the development of personalized immunotherapies tailored to the specific characteristics of the TME (**Table 1**).

**Table 1:** Cholangiocarcinoma subtypes based on the tumor microenvironment.

Year	Author	Sample (n)	Subtype and characteristic	prognosis	Reference
2012	Andersen JB	CCA (104)	1. SGI 2. SGII 3. SGIII 4. SGIV	2 > 1 > 4 > 3	(54)
2013	Sia D	iCCA (149)	1. Inflammation class (38%) 2. Proliferation class (62%)	1 > 2	(67)
2015	Nakamura H	iCCA (145) eCCA (86) GBC (29)	1. Cluster 1 2. Cluster 2 3. Cluster 3 4. Cluster 4	1 > 3 > 2 > 4	(55)
2017	Chaisaingmongkol J	iCCA (130)	1. C1 2. C2 3. C3 4. C4	3 > 2 > 4 > 1	(56)
2020	Job S	iCCA (78)	1. I1 (immune desert) 2. I2 (immune activation) 3. I3 (myeloid-enrich) 4. I4 (mesenchymal-like)	2 > 1 > 3 > 4	(60)
2020	Montal R	eCCA (189)	1. Metabolic class (18.7%) 2. Proliferation class (22.5%) 3. Mesenchymal class (47.3%) 4. Immune class (11.5%)	4 > 1 > 2 > 3	(62)
2022	Lin Y	iCCA (45)	1. The sparsely subgroup 2. The heterogeneously subgroup 3. The highly infiltrated subgroup	–	(53)
2022	Bao X	iCCA (151)	1. Subtype 1 (chronic inflammation) 2. subtype 2 (metabolism) 3. Subtype 3 (chromatin remodeling)	3 > 2 > 1	(68)
2022	Ding GY	iCCA (962)	1. Class I (immune excluded) 2. Class II (immune altered) 3. Class III (immune altered) 4. Class IV (immune active)	4 > 3 > 2 > 1	(63)
2022	Lin J	iCCA (255)	1. IG1 (immunesuppressive, 25.1%) 2. IG2 (immune-exclusion, 42.7%) 3. IG3 (immune-activated, 32.2%)	3 > 2 > 1	(57)
2022	Dong L	iCCA (262)	1. S1 (inflammatory) 2. S2 (mesenchymal) 3. S3 (metabolic) 4. S4 (differentiated)	4 > 3 > 2 > 1	(69)
2022	Chen S	iCCA (16)	1. high-immune cluster 2. low-immune cluster	1 > 2	(64)
2022	Carapeto F	iCCA (96)	1. Group 1 2. Group 2 (immune hot phenotype) 3. Group 3 (immune cold phenotype) 4. Group 4 (immune cold phenotype)	2 > 1 > 4 > 3	(61)
2023	Deng M	iCCA (114) eCCA(103)	1. S-I (metabolism subtype) 2. S-II (proliferation subtype) 3. S-III (stromal subtype)	1 > 2 > 3	(65)
2023	Cho SY	iCCA(102)	1. The stem-like subtype 2. The poorly immunogenic subtype 3. The metabolism subtype	3 > 1 > 2	(66)

In a study involving 255 human samples of iCCA (57), Lin and colleagues identified three TME-based subtypes: IG1 (immunosuppressive), IG2 (immune-exclusion), and IG3 (immune-activated). Researchers found that IG1 was characterized by excessive infiltration of neutrophils and immature dendritic cells (iDCs), whereas tumor-infiltrating T lymphocytes were predominant in IG3. Furthermore, the immune subgroups exhibited significant differences in OS and recurrence-free survival, with IG1 associated with the worst prognosis and IG3 associated with the best prognosis. Patients exhibiting an enrichment of innate immune cells within the TME may respond positively to myeloid-targeted therapies such as C-X-C motif chemokine receptor 2 (CXCR2) and colony-stimulating factor receptor (CSFR) inhibitors, which aim to deplete or reprogram tumor-associated neutrophils (58, 59). Conversely, patients with a predominance of adaptive immune cells may continue to benefit from ICI treatment. This comprehensive multimodal analysis of the three immune subgroups provides valuable insights into the immune landscape of iCCA, offering potential opportunities for personalized treatment of CCA patients. Job et al. categorized 78 human iCCA samples into four subtypes: I1 (immune desert), I2 (immune activation), I3 (myeloid-enrich), and I4 (mesenchymal-like) (60). Notably, I2 subtype exhibited a high infiltration of immune cells and demonstrated strong activation of inflammatory and immune checkpoint pathways, suggesting the potential effectiveness of immunotherapy targeting this subtype. In contrast, I4 subtype displayed the poorest overall survival, while the other two subtypes exhibited intermediate survival outcomes. This classification highlights the dynamic interaction between tumors and the immune system, aiding the identification of patients who may benefit from effective immunotherapy. Consequently, developing an immune classification method to identify CCA phenotypes characterized by high immune cell infiltration is essential to identify potential candidates for effective immunotherapy. In several other clinical studies of iCCA and eCCA have identified immune microenvironment-based prognostic subtypes, indicating a strong correlation between TILs and favorable patient outcomes (57, 61–66). Comprehensive characterization of these immune subtypes is critical for establishing CCA stratification, which may ultimately facilitate the design of subpopulation-specific immunotherapies. Concurrently, these immune subtypes with more favorable prognoses demonstrate activated inflammatory pathways (67).

Chronic inflammation within the TME may promote tumor progression, with specific immune cells linked to poor prognostic outcomes. A study identified three distinct subtypes: chromatin remodeling, metabolism, and chronic inflammation. Subsequently, Bao and colleagues found that APOE^+^ C1QB^+^ macrophage have the ability to reshape the chronic inflammation subtype, which is linked to an unfavorable prognosis in patients with iCCA (68). Another study revealed that the inflammatory subgroup characterized by high expression of inflammatory proteins and dominated by Treg infiltration exhibited a comparatively poor prognosis compared to the metabolic and differentiated subgroups. Furthermore, both inflammatory and stromal responses were found to significantly facilitate the progression of iCCA (69). Although these classifications provide a comprehensive overview of the immune landscape of CCA and suggest potential avenues for personalized treatments, no clinical applications have yet been reported. Therefore, prospective validation of these classifications is essential before they can be integrated into patient care for CCA.

## 3 Tumor-infiltrating T lymphocytes in cholangiocarcinoma

T lymphocytes originate from progenitors in the bone marrow and undergo differentiation in the thymus. Following this process, they migrate to various immune organs and tissues throughout the body via lymphatic vessels, blood, and tissue fluid circulation, where they play a crucial role in adaptive immunity (70). During the immune response, T cells can be activated and proliferate in response to specific antigens expressed by tumor cells, leading to their differentiation into effector T cells. After the immune response concludes, apoptosis occurs in the majority of effector T cells. Within the tumor microenvironment of CCA, T lymphocytes represent the most common inflammatory cell type (71). CCA can be classified into two groups based on the presence of tumor-infiltrating lymphocytes (TILs) in the TME: lymphocyte-infiltrated tumors and non-lymphocyte-infiltrated tumors (72, 73). Tumors that exhibit immune cell invasion are regarded as immune-responsive tumors, and the immune cell population in lymphocyte-infiltrated tumors can either promote or inhibit tumor progression through their immune responses (74).

The heterogeneity of tumor-infiltrating T lymphocytes is prevalent in CCA, encompassing both intertumoral and intratumoral heterogeneity (75). Intertumoral heterogeneity is closely associated with the subtype and stage of CCA, characterized by variations in the quantity, proportion, and distribution of T cells among cholangiocarcinoma subtypes (76). Goeppert observed a decreasing trend in the density of CD4^+^ and CD8^+^ T cells in the tumor immune microenvironment as bile duct cancer progressed (77). Different subsets of tumor-infiltrating T lymphocytes exert distinct effects on the long-term prognosis of CCA patients (78). Although occasional conflicting findings have been reported, infiltrated CD8^+^ T and CD4^+^ T lymphocytes are generally positively correlated with prognosis for CCA patients (71). In contrast, a high number of infiltrated Tregs may be associated with poorer overall survival. The intratumoral heterogeneity of tumor-infiltrating T lymphocytes is primarily related to T cell plasticity and intercellular communication within the TME, manifesting as state transformation and functional changes in T lymphocytes. Numerous studies have demonstrated that the TME of cholangiocarcinoma exhibits distinct T-cell states and potential trajectories for cellular development. Single-cell analyses of CCA have identified multiple subpopulations of TILs (79). A study involving 33 iCCA patients demonstrated that tissue resident memory (TRM)-like CD8^+^ TILs expressing CD69^+^ CD103^+^ showed significantly elevated levels of T cell markers (80). The phenotypic exhaustion of CD4^+^ T and CD8^+^ T cells, along with the aberrant activation of Tregs within the TME, has been extensively investigated (81). T cell exhaustion is a distinct state of cell differentiation, accompanied by changes in chromatin conformation and DNA methylation, and associated alterations in gene expression (82). Epigenetic therapy can restore defects in antigen processing and presentation of MHC-1 molecules during tumor immunoediting (83). Research has shown that increased expression of CCL5 by epigenetic treatment could increase T-cell infiltration and promote the memory and effector T-cell phenotypes (84). Additionally, the expression level of CCL5 chemokines is up-regulated, which may further attract CD8^+^ T cells to infiltrate the TME (85). Zhou and colleagues found that antibodies against TIM-3 or LAG-3 can repair the response of T cells to tumor antigens, and the combination of antibodies shows a superimposed effect (86). Heterogeneous expression patterns are also observed in certain genes both within and between different T cell subtypes. For instance, immune checkpoint molecules such as PD1, CTLA4, LAG3, and TIGIT exhibit differential expression in CD4^+^ T and CD8^+^ T cells (61). Jing and colleagues found that the expression frequency of human endogenous retrovirus-H long terminal repeat associated protein 2 (HHLA2) was higher in iCCA than in PDL1. HHLA2 overexpression is associated with a lower density of CD8^+^ TILs (87). The heterogeneous expression of these genes presents significant challenges in identifying predictors of immunotherapy responses. A comprehensive understanding of the heterogeneity among tumor-infiltrating T lymphocytes will enhance the advancement of immunotherapy.

### 3.1 CD4^+^ T lymphocytes in cholangiocarcinoma

CD4^+^ T lymphocytes play a pivotal role in the regulation of the immune system and the promotion of anti-tumor responses (88). They facilitate B cell activation for antibody production, enhance and sustain CD8^+^ T cell responses, regulate the immune response to control the strength and persistence of autoimmunity (89). These diverse functions are achieved through the differentiation of native CD4^+^ T cells upon stimulation by tumor antigens presented by antigen-presenting cells (APCs), leading to their development into effector or memory cells with specialized phenotypes (90, 91). Various subsets of CD4^+^ T cells, including Th1, Th2, Th17, and follicular T helper cells contribute differently to these processes (92, 93). Additionally, CD4^+^ T cells possess the ability to directly eliminate tumor cells by releasing cytotoxic particles (94). Recent studies also indicate the appearance of exhausted CD4^+^ T cells upon persistent antigen stimulation (95).

#### 3.1.1 The CD4^+^ T lymphocytes interact with other cells in the immune microenvironment

CD4^+^ T lymphocytes possess the ability to activate monocytes, macrophages, and NKs (96). However, CD4^+^ T cells in the TME gradually lost the ability to proliferate and recognize tumors (97). Tran and colleagues demonstrated that the progression of CCA was effectively inhibited by the adoptive transfer of T-helper (Th) cells that specifically recognize the tumor-expressed erbb2 mutant protein (98). This finding suggests that Th cell responses may facilitate the regression of late-stage CCA. The release of interleukin-10 (IL-10) by MSDCs and TAMs promotes a Th2 response while disrupting Th1/Th2 balance. Shen and colleagues found that HBV-infected iCCA patients show more Th2 cells within immune landscape (99). Studies have shown that most malignant tumors are skewed towards a Th2 response, but Qiu found that Th1 cytokines such as IFN-γ and IL-2 are mainly expressed in primary liver cancer (100). The accurate identification of T-cell phenotypes in CCA may aid in the development of effective personalized cancer immunotherapies.

#### 3.1.2 The distribution and prognosis of tumor infiltrating CD4^+^ T cells

The proportion and distribution of tumor-infiltrating CD4^+^ T lymphocytes in CCA subtypes exhibited significant disparities. Most studies have observed a marked increase in CD4^+^ T cell infiltration at the periphery of CCA compared to the central region of the tumor (76, 77, 101–103). Conversely, one study indicated that CD4^+^ T cell infiltration was notably higher at the center of the tumor than at its edge (104). Another investigation found no substantial variance in the distribution of CD4^+^ T cells surrounding and within the tumor (105). It has been demonstrated that there is a gradient decrease in T cell infiltration from the periphery to the center of the tumor, and that the total number of intraepithelial infiltrating CD4^+^ T lymphocytes serve as an independent staging and prognostic indicator for CCA (77). Kim et al. found that tumor margins with active infiltration of Foxp3^-^ CD4^+^ T helper cells exhibited higher expression levels of LAG3 and TIM3, suggesting that the infiltration of Foxp3^-^ CD4^+^ T helper cells at the tumor margin is a key group associated with clinical outcomes in patients with CCA (102). Ding and colleagues observed a significant increase in follicular helper T (Tfh) cells within the tumor and the elevated levels of Tfh cells potentially indicating a favorable prognosis (63). CCAs demonstrate diverse TILs, with a high density of CD4^+^ T cells at the tumor margin being associated with improved disease-free survival (DFS) and OS (106). Kasper et al. found that CD4^+^ T cells predominantly localize at the periphery of the tumor tissue, where they are induced by tumor cells to establish immune tolerance within the TME, thereby adapting to its immunosuppressive milieu (101). The infiltration of CD4^+^ T cells may signify malignant enhancement (**Table 2**).

**Table 2:** Distribution of tumour-infiltrating T lymphocytes in cholangiocarcinoma .

Year	Country	Author	Sample(n)	Distribution	Reference
2009	Germany	Kasper HU	CCA (8) HCC (27)	CD3^+^ T: PT > IT (*p*=0.008) CD4^+^ T: PT > IT (*p*=0.043) CD8^+^ T: PT > IT (*p ≤* 0.001)	(101)
2013	Germany	Goeppert B	pCCA (106) dCCA (43) iCCA (157) GBC (69)	CD4^+^ T: PT > IT CD8^+^ T: PT > IT Treg: PT < IT	(77)
2018	Japan	Ueno T	eCCA (117)	CD4^+^ T: PT vs IT (*p*=0.15) CD8^+^ T: PT vs IT (*p*=0.94) Treg: PT vs IT (*p*=0.62)	(105)
2019	China	Zhou G	iCCA (25) pCCA (2)	CD4^+^ T: PT > IT CD8^+^ T: PT > IT (*p*<0.001) Treg: no difference	(76)
2020	Japan	Asahi Y	iCCA (69)	CD8^+^ T: PT > IT Treg: PT > IT	(106)
2020	China	Tian L	iCCA (322)	CD8^+^ T: PT > IT (*p*<0.0001)	(117)
2021	China	Wu H	iCCA (50)	CD3^+^ T: PT > IT (*p*=0.047) CD8^+^ T: PT > IT (*p*=0.009)	(118)
2021	China	Xu YP	iCCA (140)	CD8^+^ T: PT > IT	(119)
2021	Korea	Kim HD	CCA (52)	CD4^+^ T: PT > IT (*p*<0.001) CD8^+^ T: PT > IT (*p*<0.001) Treg: PT > IT (*p*<0.001)	(102)
2021	Korea	Kim HD	iCCA (33)	CD103^+^ CD8^+^ T: PT < IT	(80)
2022	Italy	Alvisi G	iCCA (20)	CD4^+^ TRM: PT < IT (*p*<0.0001) CD8^+^ CTL: PT > IT (*p*<0.0001) CD8^+^TRM: PT < IT (*p*<0.05) Treg: PT < IT (*p*<0.0001)	(135)
2022	China	Ding GY	iCCA (39)	Treg: PT < IT (*p*<0.05) CD4^+^ Bcl6^+^ T: PT < IT (*p*<0.05)	(63)
2022	China	Xu L	eCCA (2)	CD4^+^ T: PT > IT (15.59%>3.31%) CD8^+^ T: PT > IT (34.16%>22.47%)	(103)
2023	China	Shang T	CCA (32) SL (32)	T cell: PT < IT	(78)
2023	China	Chen L	iCCA (149)	CD8^+^ T: PT vs IT (*p*=0.669) CD103^+^ CD8^+^ T: PT vs IT (*p*=0.668)	(120)
2024	China	Zhang QW	iCCA (13) SL (6)	CD4^+^ T: PT < IT (*p*<0.001) CD8^+^ T: PT > IT (*p*<0.01) γδT: PT > IT (*p*<0.05)	(104)

*HCC*, Hepatocellular carcinoma; *SL*, surrounding liver; *CD*, cluster of differentiation; *TRM*, Tissue resident memory T cells; *CD8^+^ CTL*, CD8+cytotoxic T lymphocytes; *PT*, peritumoral; *IT*, intratumoral.

### 3.2 CD8^+^ T lymphocytes in cholangiocarcinoma

CD8^+^ T cells are a subset of lymphocytes developing in the thymus and are committed to detecting antigenic peptides presented by MHC class I molecules expressed by all tumor cell types. DCs cross present the MHC class I molecules to CD8^+^ T cells to induce the generation of effector CD8^+^ T cells with cytotoxic capacity, namely CTLs (107). Following CD8^+^ T cell activation, CTLs migrate to the TME to mount effective responses (108). sustained overexpression of the receptors on CD8^+^ T cells could promote their dysfunction or exhaustion, leading to impaired efficacy in combating cancer (109). Activated CTLs employ two primary mechanisms to kill tumor cells: granule exocytosis and Fas ligand (FasL)-mediated apoptosis induction. The granule exocytosis pathway is mediated by the release of granzymes (GZM) A and B from CTLs. The released granzymes then enter cancer cells and cleave their intracellular substrates. The second mechanism is that the FasL on CTLs binds to Fas receptors on tumor cells to accelerate apoptosis (110).

#### 3.2.1 The intercellular communication of CD8^+^ T cells in the tumor microenvironment

CD8^+^ T lymphocytes play critical roles in interacting with other cells within the TME (111). As cholangiocarcinoma progresses, these interactions become attenuated. CD8^+^ T lymphocytes positively interact with immunostimulatory cells while negatively interacting with immunosuppressive cells (112). In addition to directly targeting tumor cell elimination, CTLs can also release TNF-α into the TME, inducing apoptosis in cancer cells (113). The production of prostaglandin E2 and adenosine by cholangiocarcinoma cells directly restrains the function and activity of CTLs, further inhibiting CTL-mediated anti-tumor immunity through the overexpression of immune checkpoint ligands such as PD-L1 and B7-H7, or through the downregulation of MHC-I expression on their surface (114). CAFs generate a substantial amount of extracellular matrix, impeding CTL contact with tumor cells, while also secreting the chemokine CXCL12, which inhibits T cell migration toward the tumor (115, 116). Furthermore, the activation of pathways involving TGF-β, B7-H1/PD-1, and Fas/FasL has been observed in the cholangiocarcinoma microenvironment, which hampers the proliferation and activity of CD8^+^ T lymphocytes (71).

#### 3.2.2 The distribution and prognosis of tumor infiltrating CD8^+^ T cells

Regardless of iCCA or eCCA, the predominant infiltrating inflammatory cells are CD8^+^ T lymphocytes (80, 103, 104). Numerous studies have consistently demonstrated that CD8^+^ T cells are primarily localized in the peritumor area of CCA (76, 77, 101, 102, 106, 117–119). Conversely, it has been reported that there is no significant variance in the distribution of CD8^+^ T lymphocytes around and within CCA tumors (105, 120). The quantity and positioning of CD8^+^ T cells at the tumor site are closely associated with clinical diagnosis and prognosis (121). Asahi et al. found a negative correlation between the number of CD8^+^ T cells and tumor size, suggesting that the count of CD8^+^ T cells can serve as a prognostic factor for postoperative iCCA patients (106). Immune checkpoints are closely associated with the prognosis of CCA patients. The abundant expression of ICOS, LAG3, OX40, PD-1, and TIM3 at the tumor margin indicates active participation of T cells in the immune response to tumor cells, which can lead to T cell depletion (61). Overexpression of PD-1 by CD8^+^ T cells result in the depletion of these cells and poor prognosis. Additionally, upregulated expression of the immunosuppressive cytokine IL-10 is observed in activated CD8^+^ PD-1^+^ T cells, suggesting that CD8^+^ PD-1^High^ T cells may acquire the ability to inhibit the immune response to CCA (117) (**Table 2**).

### 3.3 Tregs of tumor microenvironment in cholangiocarcinoma

Regulatory T cells, commonly referred to as Tregs, represent a subset of CD4^+^ T cells within the immune system characterized by low proliferation capacity and typically expressing phenotypes such as CD4^+^ CD25^+^ Foxp3^+^ and CD4^+^ CD25^+^ CD127^low^ (122). Tregs can be categorized into thymus-derived Tregs (tTregs) and peripherally-derived Tregs (pTregs) (123, 124). Tregs are capable of secreting cytokines such as IL-4, IL-10 and TGF-β, which contribute to the maintenance of immune homeostasis by regulating immune responses within the organism. The transcription factor Foxp3, specifically expressed by Tregs, plays an essential role in their maturation and function (125). Recent studies have revealed that Tregs not only participate in immunosuppressive regulation but also play a substantial role in tumor immune evasion (126, 127).

#### 3.3.1 The cross-talk between Tregs and other cells within tumor microenvironment

Tregs in cholangiocarcinoma often work with immunosuppressive cells to promote tumor progression and inhibit CD4^+^ T and CD8^+^ T lymphocytes activity in TME in a variety of ways (128). Tumor cells, TAMs, and CAFs release CCL22/CCL17 to bind to CCR4 on the surface of Tregs, and recruit a large number of Tregs to move to the TME (129). CTLA-4 is highly expressed on Tregs and competitively binds to CD80 and CD86 with T cells, leading to a reduction in T cell proliferation and cytokine production. In addition, Tregs down-regulated the function of DCs by competitively binding CD80 (130). Treg produces IL10 and TGF-β, attracts more immunosuppressive cells, transforms DCs into regulatory dendritic cells that produce indoleamine 2, 3-dioxygenase, and blocks the immune system’s rejection of cancer (131). In a study, researchers found that *MUC1* interacts with the epidermal growth factor receptor (EGFR) and its downstream carcinogenic pathway EGFR/PI3K/Akt is activated, leading to the accumulation of Tregs. This accumulation enhances the malignant phenotype of CCA cells and ultimately promotes their metastasis and growth (132). Additionally, *FoxM1* binds to the Foxp3 promoter region and promotes FoxP3 transcription. The overexpression of *FoxM1* enhanced the inhibitory effect of Treg cells on CD8^+^ T cytotoxicity, promoting immune escape in cholangiocarcinoma (133). Notably, a study demonstrated that knockdown of FoxP3 reduces the proliferation and invasiveness of CCA cells (134).

#### 3.3.2 The distribution and prognosis of tumor infiltrating Tregs in cholangiocarcinoma

There is currently no consensus regarding the spatial distribution of Tregs. Zhou and colleagues reported the presence of Tregs both within the tumor and at the tumor margins in iCCA and pCCA. Their study also noted an enhanced expression of CD69, an activation marker for Tregs, suggesting that CCA has immunosuppressive microenvironment characteristics (76). Similarly, Ueno observed a consistent distribution of Tregs in and around eCCA tumors (105). A study involving 52 patients receiving palliative gemcitabine in combination with cisplatin for BTC showed that the density of Tregs was significantly higher at tumor margins compared to interstitial and core areas. However, Treg density did not correlate with PFS and OS (102). Asahi also reported more Treg infiltration at the tumor margins in iCCA (106). In contrast, Alvisi and colleagues provided a comprehensive analysis of various lymphocyte subsets present in iCCA patients, revealing extensive aggregation of Tregs with a highly immunosuppressive phenotype within tumors (135). Ding (63) and Goeppert (77) similarly noted that higher densities of Tregs in CCA were found in the tumor core region. In addition, Goeppert and colleagues found that a gradual decrease in Tregs was associated with tumor aggressiveness and metastasis. Meanwhile, compared to patients with lower Treg counts in tumor tissue, those with higher Treg counts exhibited a better prognosis, suggesting that the immunosuppressive effect of Tregs may not be a major factor in progression of BTC (77) (**Table 2**).

## 4 The immune checkpoints in cholangiocarcinoma

Tumor cells interact with surrounding cells to create a microenvironment that supports their growth and development while evading immune surveillance through various mechanisms, thereby achieving immune escape (136). Traditional treatments for CCA, such as chemotherapy and radiation, often lack specificity, leading to indiscriminate attacks on immune cells and subsequent immune system disorders (137). Immunotherapy aims to enhance or restore the ability of autoimmune cells to recognize and eliminate tumor cells, which is more in line with the anti-tumor mode of the body. Current immunotherapy approaches for CCA primarily focus on T lymphocytes. However, the complexity of the CCA immune microenvironment results in inconsistent responses to anti-tumor therapies, presenting both opportunities and challenges for the development of personalized immunotherapy (138).

**Figure 3:**
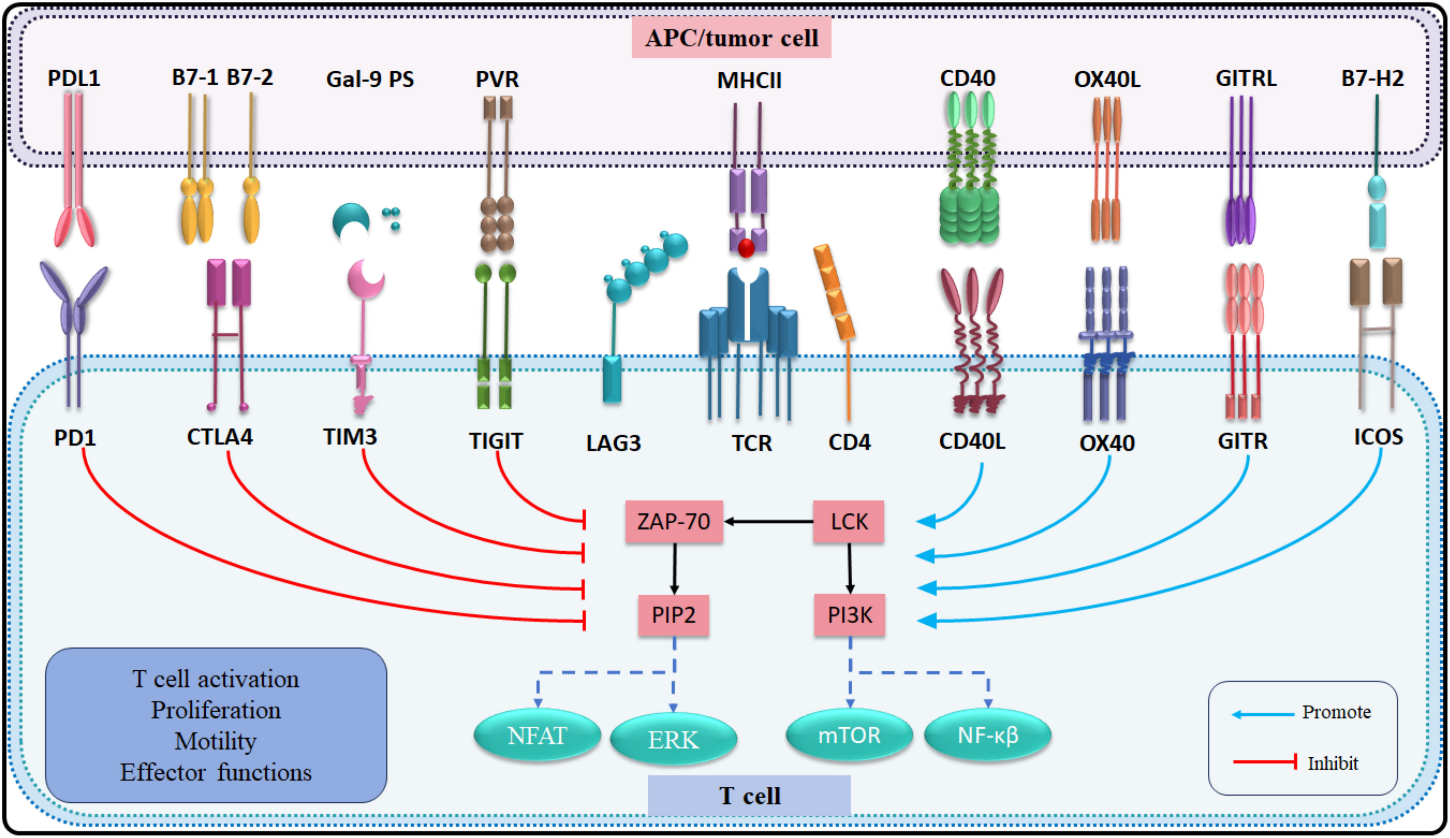
List of the immune checkpoints and their receptors in CCA.

The immune response of T lymphocytes is regulated not only by antigen-specific signals but also by numerous immune checkpoint signaling pathways (139). In recent decades, therapies targeting immune checkpoints have emerged as a promising approach to immunotherapy (140). Co-stimulatory immune checkpoints such as CD40L, OX40, GITR, and ICOS enhance cell activation, whereas co-inhibitory immune checkpoints, including PD-1, CTLA-4, TIM-3, TIGIT, and LAG-3 negatively regulate immune cell activation. The clinical application of blocking co-inhibitory immune checkpoints or activating co-stimulatory immune checkpoints has demonstrated significant potential in the treatment of advanced CCA. The presence of multiple co-expressions on T cells suggests that combination therapy targeting different immune checkpoints may yield more effective therapeutic outcome compared to single immune checkpoint therapies (141) (**Table 3**; **Figure 3**).

**Table 3:** Immune checkpoints in cholangiocarcinoma.

Acceptor	Other name	Mainly expressed	Ligand	Other name	Mainly expressed	Immune
PD-1	CD279	immune cells TILs	PD-L1 PD-L2	CD274	Tumor cells APCs	(-)
CTLA-4	CD152	activated T cells Tregs	B7-1 B7-2	CD80 CD86	APCs	(-)
TIM-3	CD366 HAVCR2	activated CD4^+^ T cells	Gal-9 PtdSer HMGB1 CEACAM-1			(-)
TIGIT	WUCAM Vstm3 VSIG9	activated T cells activated NKs	CD155 CD112 CD113	PVR Nectin-2 PVRL3	Tumor cells APCs	(-)
LAG-3	CD223	activated CD4^+^ T activated CD8^+^ T TILs	MHC II Gal-3 LSECtin FGL-1		APCs	(-)
CD40	TNFRSF5 Bp50	APCs	CD40L	CD154 TRAP gp39 TNFSF5	activated CD4^+^ T cells	(+)
OX40	CD134 ACT35 TNFRSF4	activated Tregs Activated NKT cells	OX40L	CD252 TNFSF4 CD134L gp34	B cells DCs	(+)
GITR	TNFRSF18 CD357 AITR	Tregs effector T cells	GITRL		activated APCs	(+)
ICOS	CD278	activated T cells	ICOSL	B7-H2	APCs	(+)

The “-” in the table represents the co-inhibitory immune checkpoints, the “+” represents the co-stimulatory immune checkpoints.

### 4.1 PD-1

Programmed cell death protein-1 (PD-1) is a transmembrane protein widely expressed in various activated immune cells. When PD-1 binds to programmed death-ligand 1 (PD-L1) on the surface of tumor cells, the immunoreceptor tyrosine-based inhibitory motif (ITIM) and immunoreceptor tyrosine-based switch motif (ITSM) of PD-1 are phosphorylated (142). Subsequently, Src homology region 2 domain-containing phosphatase (SHP-2) is recruited and activated, inhibiting the phosphorylation of downstream signaling of TCR and CD28. As a result, PD-1 inhibits the activation, proliferation, and cytotoxic secretion of T cells in cancer. The PD-1/PD-L1 pathway plays a crucial role in maintaining immune tolerance within the tumor microenvironment and facilitating the immune escape of tumor cells (143). PD1/PDL1 has emerged as a significant clinical biomarker for prognosticating the effectiveness of immunotherapy in solid tumors (144). At present, antibodies blocking PD-1 or its ligand PD-L1 have been approved to treat various solid and hematologic malignancies (145).

Tian and colleagues found that iCCA patients exhibiting a high proportion of CD8^+^ PD-1^High^ T cells had worse postoperative survival compared to those with a low proportion of these cells. Furthermore, a high proportion of tumor-infiltrating CD8^+^ PD-1^High^ T cells was significantly correlated with advanced TNM stage (117). This finding suggests that a high percentage of CD8^+^ PD-1^High^ T cells may serve as an independent prognostic factor. However, it is important to note that the data for this study were derived from a single hepatobiliary center, and no prospective studies have been conducted to validate these results. Previous studies have demonstrated the expression of PD-1/PD-L1 in CCA and its correlation with ICI treatment response, the predictive value of PD-L1 in CCA remains uncertain. A clinical trial using pembrolizumab for various advanced cancers included 104 patients with BTC. Although results showed that the objective response rate (ORR) was slightly higher in patients with positive PD-L1 expression than in patients lacking PDL1 expression, significant differences in median PFS or OS were not observed (146). This limited response to monotherapy with an immune checkpoint inhibitor in an unselected cohort of advanced BTC underscores the necessity of identifying specific biomarkers and screening patients who may response from treatment. In another clinical trial that included a cohort of 20 patients with advanced solid tumors, 23 BTC patients with positive PD-L1 expression had a 17% ORR, a median PFS of 1.8 months, and a median OS of 6.2 months after receiving pembrolizumab. The highest response rates were found in patients with elevated tumor mutational burden and inflammatory markers (GEP or PD-L1) (147). These results suggest that a combination of biomarkers may help identify patients most likely to respond to ICIs, while also indicating that it may be feasible to enhance the antitumor response through combination therapy.

### 4.2 CTLA-4

Cytotoxic T lymphocyte antigen 4 (CTLA-4) is a member of immunoglobulin related receptors family and is predominantly found in intracellular vesicles in Tregs or activated conventional T cells (148). This localization is due to the constitutive endocytosis of the plasma membrane and results in 90% of CTLA-4 being intracellular. CTLA-4 competes with CD28 to bind two different ligands of APC, CD80 and CD86, to regulate adaptive immune responses and inhibit T cell overactivation (149). CTLA-4 tends to have an advantage due to CTLA-4 interacts with both ligands with higher affinity and avidity than CD28. Blocking CTLA-4 is capable of generating an immune response to cancer and self-tissue, and targeting the CD28/CTLA-4 pathway with antibodies has shown considerable promise in the treatment of cancer and autoimmune diseases. Experiments have shown that anti-CTLA-4 therapy combined with Treg consumption is more effective in inducing anti-tumor response than blocking CTLA-4 alone (150), reducing tumor Infiltrating Treg may be an important factor in determining immunotherapy response.

A study found that CTLA-4^+^ lymphocyte density was elevated in iCCA compared with peritumoral hepatic tissues, and patients with a high density of CTLA-4^+^ tumor-infiltrating lymphocytes (TILs^CTLA-4 High^) showed a reduced OS compared with patients with TILs^CTLA-4 Low^ (151). Clinically, the density of CTLA-4^+^ TIL serves as an independent risk factor for evaluating OS in patients with iCCA. Additionally, the expression of CTLA-4 in TILs is critical in maintaining an inhibitory immune microenvironment in iCCAs. Another study showed a different outcome, the elevated density of CD4^+^ or CD8^+^ TILs in patients with high CTLA-4 expression on interstitial lymphocytes or tumor cells, the superior outcomes in the group of high CTLA-4 expression level (152). This study underscores the potential prognostic significance of CTLA-4 expression in eCCA. Notably, the impact of CTLA-4 expression on survival appears to vary depending on the tumor location. This study is not without limitations, its retrospective design and relatively small patient cohort necessitate the acquisition of additional datasets to verify the reliability of the findings. In a study involving 20 patients with advanced BTC, participants received tremelimumab in conjunction with radiofrequency ablation. The results indicated that two patients achieved partial response, and five patients achieved stable disease. Furthermore, an analysis of the cell subsets in these patients post-treatment revealed an increase in the number of activated CD8^+^ T cells in peripheral blood (153). Although this small study yields limited conclusions, a correlation between immune markers and clinical responses appears to exist.

### 4.3 TIM-3

T cell immunoglobulin and mucin domain 3 (TIM-3) was originally found to be expressed on the surface of Th1 cells, Expression of TIM-3 on CD8^+^ T cells in the tumor microenvironment is considered a cardinal sign of T cell dysfunction. Recent studies have shown that TIM-3 is also expressed on other immune cells (154). TIM-3 binding to the ligand galectin-9 to mediate Th1 cell the apoptosis. Tregs highly express TIM-3 and secrete IL-10 to inhibit the function of effector T cells in the TME (155, 156). TIM-3 has emerged as an important checkpoint molecule whose expression correlates with to promote T cell exhaustion in chronic viral infection and cancer (157). A number of clinical trials are under way using blocking monoclonal antibodies directed against TIM-3, however the exact mechanisms underlying the anti-tumor activity of these antibodies are not well understood. In CCA, the highly immunogenic iCCA expressed high levels of TIM-3 (141). Furthermore, TIM-3 is upregulated in infiltrating CD8^+^ T and infiltrating CD4^+^ T cells, and high expression of TIM-3 in CD8^+^ T cells is associated with lymph node metastasis of iCCA (158). A study demonstrated that reducing the expression of several inhibitory molecules, including TIM-3, in CAR T cells resulted in robust immunity against CCA, exhibiting long-term efficacy both *in vitro* and *in vivo* (159). However, the safety and efficacy of this approach require further validation through preclinical trials.

### 4.4 TIGIT

T cell immunoreceptor with immunoglobulin and ITIM domain (TIGIT) is a receptor of the Ig superfamily, expressed by activated CD8^+^ T and CD4^+^ T cells in humans, which potently inhibits innate and adaptive immunity through multiple mechanisms (160). TIGIT binds to two ligands, CD155 and CD112, that are expressed by tumor cells and antigen presenting cells in the tumor microenvironment. TIGIT indirectly impedes T cell function by binding to CD155 on DCs. Second, TIGIT exhibits direct immune cell-intrinsic inhibitory effects (161). Dmitrij and colleagues found that TIGIT was upregulated in tumor-infiltrating CD8^+^ T cells. Furthermore, TIGIT can accurately identify exhausted CD8^+^ T cells at various stages of differentiation (162). Nicole found that TIGIT is highly expressed by Tregs in the peripheral blood mononuclear cells of healthy donors and cancer patients, and is further upregulated in the TME (163). There is now strong evidence that TIGIT regulates both T-cell-mediated and natural killer cell-mediated tumor recognition *in vivo* and *in vitro*. Dual PD-1/TIGIT blocking enhances *in vitro* expansion and function of tumor antigen-specific CD8^+^ T cells and promotes tumor rejection in mouse tumor models (164, 165). These findings support the development of ongoing clinical trials of PD-1/TIGIT dual blocking to treat cancer patients.

### 4.5 LAG-3

Lymphocyte activation gene 3 (LAG-3) is an inhibitory receptor that is highly expressed by exhausted T cells (166). While LAG-3 negatively regulates T cell activation and function, its significance in other cell types remains unclear. LAG-3 is widely expressed by many cell types of both lymphocytic and nonlymphocytic lineage and its expression is a hallmark of exhausted CD4^+^ T and CD8^+^ T cells in the context of persistent antigenic stimulation by tumors or chronic viral infections (167)LAG-3 has a higher binding affinity with its typical ligand major histocompatibility complex (MHC) class II than CD4. which can competitively bind MHCII with CD4 and inhibit CD4^+^ T cell function (168). LAG-3 is a promising immunotherapeutic target, with more than 20 LAG-3-targeting therapeutics in clinical trials (169). The immune profiling analysis of peripheral blood reveals an increased abundance of LAG-3^hi^PD-1^hi^ memory CD4^+^ T cell subset in relapsed cholangiocarcinoma patients after gemcitabine plus cisplatin therapy, which provided a basis for the study of immune checkpoint inhibitors for CCA (170). In addition, the study demonstrated that bispecific antibodies targeting LAG-3 and PD-L1 elicit an effective anti-tumor response from immune cells in the tumor microenvironment, although these results further support the potential of targeting LAG-3 as a cancer immunotherapy. However, further research is needed to explore the modulation of tumor-infiltrating T lymphocytes and its translational value.

### 4.6 OX40

Tumor necrosis factor receptor superfamily member 4 (TNFRSF4), also known as OX40, is a type 1 transmembrane glycoprotein predominantly expressed by activated T lymphocytes (171). The cytoplasmic domain of OX40 is involved in downstream signaling pathways by binding to the tumor necrosis factor receptor-associated factor family (TRAF) of intracellular proteins. Its ligand OX40L, which also belongs to the tumor necrosis factor superfamily, is primarily expressed on APCs. The interaction between OX40 and OX40L has immunomodulatory function on T cell survival and proliferation. Studies have shown that increased OX40 expression in CD8^+^ T cells with IL-2 via STAT5-mediated signaling in the setting of weak TCR stimulation (172). OX40 signaling also reduces the expression of FOXP3 and CTLA-4, leading to a decline in Tregs function. Furthermore, activated T cells exhibit increased expression of CD28 and enhanced expression of OX40 (173). Giampietri and colleagues found that OX40 was significantly upregulated in 36 cholangiocarcinoma samples compared to 9 normal control samples, suggesting that OX40 may play a potential role in cholangiocarcinoma, as both a diagnostic or prognostic marker and a therapeutic target (174). Another study showed that the frequency of OX40^+^ nTregs (naïve Tregs) and OX40^+^ eTregs (effector Tregs) in peripheral blood of CCA patients was significantly higher than that of healthy controls, both before and after surgery (175). This suggests that OX40^+^ nTregs (naive Tregs) and OX40^+^ eTregs can be used as biomarkers of therapeutic effect and prognosis of CCA.

### 4.7 CD40L

Cluster of differentiation 40 ligand (CD40L) is a 39-kDa type II transmembrane protein. The expression of CD40L is typically inducible and primarily restricted to cells of the hematopoietic system (176). CD40 can bind to its ligand CD40L, which activates dendritic cells, enhances antigen presentation, and activate T cells by up-regulating the expression of co-stimulatory molecules while down-regulating immunosuppressive molecules (177). CD40/CD40L immune checkpoint leads to activation of both innate and adaptive immune cells via two-way signaling. CD40/CD40L interaction also participates in regulating thrombosis, tissue inflammation, hematopoiesis and tumor cell fate. *In vitro* experiments have demonstrated that specific blockade of tumor-secreted IL-10 and TGF-β can lead to the up-regulation of CD40, thereby enhancing the cytolytic activity of effector T cells against CCA cells (178). Current evidence suggests that immunotherapy for CCA holds promise through the activation of CD40/CD40L immune checkpoints (179). An animal demonstrated that combination therapy with a CD40 agonist resulted in a superior effector response compared to anti-PD-1 monotherapy for CCA, accompanied by an increased presence of CD4^+^ T and CD8^+^ T cells in tumor-bearing mice (180). Immunotherapy for CCA by activating CD40/CD40L immune checkpoints is a promising approach. Second, strong expression of CD40 was observed in tumor samples from half of patients with cholangiocarcinoma. However, the effects observed in this study were not associated with positive expression of CD40 in tumor cells. It is possible that other factors such as the expression of cytokines IFN-γ or TNF-α are also involved in the process of inducing apoptosis of tumor cells.

### 4.8 GITR

Glucocorticoid-induced tumor necrosis factor receptor-related protein (GITR) is a member of the TNF receptor superfamily, consistently expressed at high levels on the surfaces of Tregs (181). GITR ligand (GITRL) is mainly expressed in dendritic cells, B cells, macrophages, and endothelial cells. APCs not only constitutively express GITR ligand (GITRL) but also enhance its expression under stimulating conditions (182). The signaling mediated by GITR plays a crucial role in regulating immune responses by providing costimulatory signals that enhance responder T cell functions such as activation, differentiation, survival, and memory formation while simultaneously counteracting the immunosuppressive effects of Tregs (183). Blockade of the GITR/GITRL system has proven beneficial in treating autoimmune diseases and in transplantation, whereas stimulation with an agonistic antibody has reversed immunosuppressive responses in chronic infections and tumors (184). Zhou found that activating GITR on T cells within cholangiocarcinoma tumors increased their production of effector molecules and proliferation, suggesting that targeting GITR could be a potential immunotherapy for CCA patients (76). However, the limited cohort of patients in this study did not correlate immunological data with patient survival, indicating that the clinical application of these findings requires further validation.

### 4.9 ICOS

Inducible Co-Stimulator (ICOS) is predominantly expressed on activated T cells (185). Its ligand ICOSL is expressed on antigen-presenting cells and somatic cells, including tumor cells in the tumor microenvironment. The expression of both ICOS and ICOSL correlates with the release of cytokines that are induced by immune response activation (186). Together, ICOS and ICOSL facilitate a range of activities across various T cell subsets, encompassing T cell activation, effector functions, and the inhibitory activities mediated by Tregs (187). This dual role in both antitumor and protumor activity makes targeting the ICOS/ICOSL pathway attractive for enhancing antitumor immune responses. A study indicated that ICOS expression is elevated in the TME of cholangiocarcinoma, particularly in regions with increased extracellular matrix distribution, which has significant implications for the stratification of immunotherapy (141). Additionally, Carapeto reported that ICOS expression is greater at the tumor margin compared to the tumor center, and low ICOS expression in iCCA is associated with poor OS (61). These studies also demonstrated the co-expression of checkpoint molecules in CCA, suggesting the necessity for combined therapy targeting different immune checkpoints. However, it remains unclear whether immune cells can be recruited into the immune microenvironment when ICIs are used to treat CCA. Furthermore, investigating the distribution of checkpoint molecules may be crucial in determining the optimal treatment strategies for patients receiving combinations of chemotherapy and immunotherapy.

### 4.10 Combination therapy for immune checkpoints

Given the limitations of ICI monotherapy, there is significant interest in developing combined immunotherapy strategies. A comprehensive analysis of a database comprising 290 iCCA patients, alongside tumor tissue immunohistochemistry, revealed that CTLA-4^+^ TILs and PD-L1^+^ TILs can independently predict tumor recurrence and OS in iCCA patients following surgical resection (151). Consequently, therapies targeting both PD-1/PD-L1 and CTLA-4 may offer potential advantages for the treatment of iCCA patients. The premise of the dual ICI is that blocking a single checkpoint may not be enough to activate CTLs. In a Phase 2 study evaluating the combination of nivolumab and ipilimumab in 39 patients with advanced-stage BTC, the trial reported an overall response rate (ORR) of 23% and a disease control rate of 44% (188). These findings highlight the potential superiority of dual ICI combination therapy compared to monotherapy. Notably, these responses were observed exclusively in patients with iCCA or gallbladder cancer. In developing effective immune-specific therapeutics, understanding the immune landscape characteristics of each CCA subtype will be crucial (189). Although the combination of CTLA-4 and PD-1 blockade improved efficacy, it also increased the incidence of adverse events (AE) in CCA. Results from a clinical study indicated that the early treatment outcomes of durvalumab combined with tremelimumab in patients with hepatocellular carcinoma and BTC were relatively disappointing, particularly among BTC patients. In the BTC cohort, the median progression-free survival was 3.1 months, and the overall survival was 5.5 months. Additionally, multiple grade 3/4 treatment-related adverse events were reported (190). Given the heightened risk of AE and the limited efficacy of PD-1/CTLA-4 blockade, there is considerable interest in exploring alternative combination immunotherapies.

Two clinical trials are currently underway targeting CD40 (NCT03329950) and OX40 (NCT03071757) as monotherapy or in combination therapy for advanced cancers including BTC (191), Additionally, investigations are underway into combination strategies involving ICIs alongside other treatments for CCA, in addition to ICI immunotherapy administered alone. These strategies include combined local ablation, radiotherapy, intra-tumor injection, and chemotherapy, all of which aim to enhance tumor antigen exposure and thereby increase the likelihood of an immune response when used in conjunction with ICIs (192, 193). The combination of ICIs with anti-angiogenic therapy has the potential to promote immune responses by suppressing immunosuppressive factors or by increasing immune cytokines within the TME (194). Finally, combinations of various immune checkpoint inhibitors with standard chemotherapy have shown an acceptable safety profile in several early-stage clinical trials (195).

## 5 Conclusions and future directions

CCA is a malignant tumor characterized by its insidious onset and poor prognosis. At present, the main treatment modalities for CCA have not been effective in reducing patient mortality. Immunotherapy, as a novel treatment approach for solid tumors, offers significant hope for CCA patients. However, the application of immunotherapy in CCA encounters substantial challenges. Firstly, CCA is a heterogeneous disease, and the immune microenvironment varies among patients, which affects the efficacy of immunotherapy. Secondly, most CCA patients present with immunosuppressive microenvironments, resulting in low response rates to immunotherapy. Lastly, treatment with a single ICI has shown limited effectiveness, and patients are often prone to developing resistance. These challenges underscore the need for a deep understanding of the immune landscape of CCA, a comprehensive assessment of patient immune status, and the development of personalized combination immunotherapy regimen to address the therapeutic difficulties posed by the immune diversity of CCA. Current immunotherapy strategies primarily target T lymphocytes within the TME, with a particular emphasis on CD8^+^ T cells. Tumor-infiltrating T cells display various phenotypes and functional states, and the heterogeneity among these T lymphocytes is linked to the malignant progression of CCA as well as the effectiveness of immunotherapy. To achieve more effective and precise treatments, future research should utilize single-cell and multi-omics to explore the complex mechanisms of T-cell interactions with CCA. This could facilitate the development of novel immunomodulators and herald a new era in CCA therapy.

Combination therapy focusing on ICIs has emerged as the first-line treatment for CCA. Dual ICI therapy, which targets different immune checkpoints, has also demonstrated the anticipated synergistic therapeutic effect. However, it is important to note that in clinical practice, most patients exhibit resistance to ICI combination treatment, leading to poor overall prognosis and relapse-free survival rates. Consequently, there is an urgent need to develop a stratified treatment model for CCA patients and to identify biomarkers that can facilitate more accurate and reliable immunotherapy. Additionally, it is essential to evaluate the safety and adverse reactions associated with ICI combination therapy. This necessitates further high-quality, prospective, and randomized controlled trials to ascertain the safety and therapeutic efficacy of various immunization combination regimens. From a clinical perspective, it is vital to consider both standard treatment regimens and the optimal combinations with immunotherapy. On the immunotherapeutic front, ongoing clinical trials are investigating novel immune checkpoint therapies. The study of tumor-infiltrating T cells in CCA has thus far shown promising potential for advancing immunotherapy strategies. With these emerging therapeutic options, patient prognosis in CCA is anticipated to improve.
